# Modeling the Endothelial Glycocalyx Layer in the Human Conventional Aqueous Outflow Pathway

**DOI:** 10.3390/cells11233925

**Published:** 2022-12-04

**Authors:** Alireza Karimi, Mahdi Halabian, Reza Razaghi, J. Crawford Downs, Mary J. Kelley, Ted S. Acott

**Affiliations:** 1 Department of Ophthalmology and Visual Sciences, University of Alabama at Birmingham, Birmingham, AL 35233, USA; m.halabian93@gmail.com (M.H.); razaghireza@outlook.com (R.R.); cdowns@uabmc.edu (J.C.D.); 2 Departments of Ophthalmology and Integrative Biosciences, Casey Eye Institute, Oregon Health & Science University, Portland, OR 97239, USA; kelleyma@ohsu.edu; 3 Departments of Ophthalmology and Biochemistry and Molecular Biology, Casey Eye Institute, Oregon Health & Science University, Portland, OR 97239, USA; acott@ohsu.edu

**Keywords:** endothelial glycocalyx layer, trabecular meshwork, juxtacanalicular tissue, Schlemm’s canal, aqueous outflow resistance, electro-fluid–structure interaction

## Abstract

A layer of proteoglycans and glycoproteins known as glycocalyx covers the surface of the trabecular meshwork (TM), juxtacanalicular tissue (JCT), and Schlemm’s canal (SC) inner wall of the conventional aqueous outflow pathway in the eye. This has been shown to play a role in the mechanotransduction of fluid shear stress and in the regulation of the outflow resistance. The outflow resistance in the conventional outflow pathway is the main determinant of the intraocular pressure (IOP) through an active, two-way, fluid–structure interaction coupling between the outflow tissues and aqueous humor. A 3D microstructural finite element (FE) model of a healthy human eye TM/JCT/SC complex with interspersed aqueous humor was constructed. A very thin charged double layer that represents the endothelial glycocalyx layer covered the surface of the elastic outflow tissues. The aqueous humor was modeled as electroosmotic flow that is charged when it is in contact with the outflow tissues. The electrical–fluid–structure interaction (EFSI) method was used to couple the charged double layer (glycocalyx), fluid (aqueous humor), and solid (outflow tissues). When the IOP was elevated to 15 mmHg, the maximum aqueous humor velocity in the EFSI model was decreased by 2.35 mm/s (9%) compared to the fluid–structure interaction (FSI) model. The charge or electricity in the living human conventional outflow pathway generated by the charged endothelial glycocalyx layer plays a minor biomechanical role in the resultant stresses and strains as well as the hydrodynamics of the aqueous humor.

## 1. Introduction

The aqueous humor outflow resistance in the conventional outflow pathway is the primary determinant of intraocular pressure (IOP) [1,2,3,4]. Impairment in providing a dynamic balance between the aqueous humor inflow and outflow due to an abnormally high outflow resistance in the conventional outflow pathway results in an IOP elevation that is associated with primary open angle glaucoma (POAG) [5,6,7,8,9,10,11,12]. Aqueous exits the eye through the conventional (>70%) and unconventional (<30%) outflow pathways. Aqueous humor passing through the conventional outflow pathway encounters the trabecular meshwork (TM), the juxtacanalicular tissue (JCT), and the inner wall endothelium of Schlemm’s canal (SC). The aqueous humor then enters the SC lumen and flows circumferentially to reach collector channels leading to the aqueous and episcleral veins [13,14,15,16,17].

As in vascular endothelial cells, an extracellular matrix layer of proteoglycans and glycoproteins known as glycocalyx with an average thickness of 52–166 nm [18], covers the TM, JCT, and SC cells, the pores in the SC inner wall, as well as the inner membrane of giant vacuoles [14,18,19,20,21,22] in the human conventional outflow pathway. The glycocalyx layers function as sensors of fluid shear stress [23,24,25] and mediate the alignment of endothelial cells in the direction of flow [26,27,28]. It significantly contributes to the initiation of responsive actions in the actin network of the cytoskeleton via mechanosensation [28,29]. One part of mechanotransduction is through the stimulation of endothelial nitric oxide synthase (eNOS), which drives the release of nitric oxide (NO) [30,31,32]. In the SC inner wall, the glycocalyx layer plays a key role in sensing and responding to the fluid shear stress, mainly through the shear-induced eNOS activity and contributes to rapid outflow resistance regulation [14,33,34,35]. Releasing the NO in the outflow pathway due to the fluid shear stress also results in increasing the outflow facility [21] and endothelial permeability [25,36,37]. In healthy eyes, the glycocalyx layer has a variable distribution that may contribute to the segmental (low- and high-flow [38,39]) flow of aqueous humor [40,41], which is analogous in some ways to the vascular system as a result of heterogeneous blood flow distribution [42,43]. There is an electrical charge in living human tissues, especially the eye [44], and aqueous humor is an electrical conductor because of its salinity [45]. However, we are not aware of any experimental or numerical studies that have calculated the biomechanical contribution of the glycocalyx layer in the outflow resistance and IOP regulation across the outflow pathway. Experimentally speaking, it would be difficult with current imaging techniques to measure the outflow resistance in vivo; numerical techniques can help to overcome this limitation. 

In prior studies, our group has calculated the elastic and viscoelastic biomechanical properties of the healthy and glaucomatous human outflow tissues using dynamic SC pressurization, high-resolution optical coherence tomography (OCT), and finite element method coupled with an optimization algorithm [46,47,48]. Through an active, two-way, fluid–structure interaction of the outflow tissues and aqueous humor dynamics, we studied the hydrodynamics of the aqueous humor across the healthy and glaucomatous outflow pathways [49,50]. While we have studied the biomechanics of the human aqueous outflow pathway using experimental and computational techniques, the important role of the glycocalyx layer in the resultant outflow resistance across the outflow pathway has not been fully explored. In our prior study, we developed an analytical approach to model the glycocalyx layer in the blood vessel using a very thin electrically charged double layer [51]. Our results indicated that while the interaction of the negatively charged glycocalyx layer and the positively charged blood flow affects the hemodynamics of the blood in a vessel, the biomechanical role of the glycocalyx in the blood vessel is minor. In this study, a 3D microstructural finite element model of the outflow pathway, including the TM, JCT, and SC inner wall, with interspersed aqueous humor was constructed. The model was subjected to an IOP elevation of 0 to 15 mmHg from the anterior chamber. A very thin electric double layer that represents the glycocalyx layer based on our prior study [51] with a uniform thickness of ~109 nm [19] was added to the outflow tissues’ surfaces. The aqueous humor and outflow tissues were also charged positively and negatively, respectively, through the electroosmotic flow generated by the interaction of the moving aqueous humor with the outflow tissues. The glycocalyx layer (electric), aqueous humor (fluid), and outflow tissues (solid) were coupled using an electrical–fluid–structure interaction (EFSI) method. The EFSI equations in the modeled outflow tissue with complex geometries were solved using COMSOL Multiphysics (COMSOL, Inc., MA USA). The results were interpreted as the stresses and strains in the outflow tissues as well as the hydrodynamics of the aqueous humor across the outflow pathway. 

## 2. Materials and Methods

### 2.1. Finite Element Reconstruction, Volume Meshing, Material Models, and Boundary Conditions

The 3D FE model of the TM/JCT/SC complex of a normal human donor of European descent was constructed [52]. The descriptions in regard to the imaging, segmentation, and volume meshing of the TM/JCT/SC complex FE model were fully explained in our prior publications [46,47,49,50,53].

**Figure 1:**
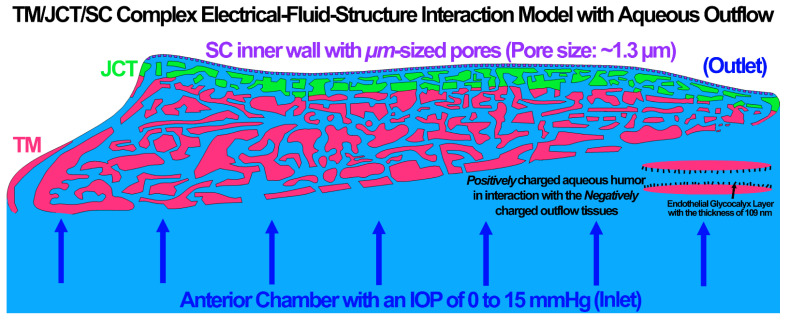
The electrical–fluid–structure interaction model of the TM, with adjacent SC (~2.2 µm) and JCT (~14 µm), and aqueous humor. The µm-sized pores with the average size of ~1.3 µm and the density of 835 pores/mm^2^ were distributed in the SC inner wall.

The model was volume meshed [54] and separated into the TM, with adjacent JCT (~14 μm [55]) and SC inner wall including its greater basement membrane (~2.2 μm [56]) regions as shown in Figure 1. Micrometer-sized pores were distributed in the SC inner wall [57], with a pore density and diameter of 835 pores/mm^2^ [56] and 1.3 µm [58], respectively (Figure 1). The models were meshed with 8-noded hexahedral elements [59], including 348,861 elements and 433,636 nodes, and the element edge length of ~0.5–1 μm. Element quality assessment and time-step analysis were carried out using LS-DYNA (Ansys/LS-DYNA, Pennsylvania, US) [48,54,60,61,62]. Mesh density analyses were conducted for the FE model as described in our previous publications [46,47,49,50]. To have a fully unbiased analysis, the structure, density, and distribution of the mesh in the models were the same.

The outflow tissues were treated as elastic materials with the moduli of 4 [63], 4 [63], and 7.48 kPa [64] for the TM, JCT, and SC inner wall, respectively. The tissues were assumed nearly incompressible with Poisson’s ratio of 0.495. The electric potential of the endothelial cells that covered the outflow tissues was −70 mv with the reference impedance of 50 Ω [65,66].

A pre-tension force of ~500 μN was generated in the TM/JCT/SC complex local nodes to mimic the ciliary muscle movement during IOP fluctuation that also helps to prevent sudden excessive dynamic response in the cables [17,67].

### 2.2. Electrical–Fluid–Structure Interaction

In our prior study, we developed an analytical approach to model the glycocalyx layer in the blood vessel using a very thin electric double layer [51]; however, herein, due to the complexity of the outflow tissues’ geometry the same approach cannot be applied. Thus, in this study, the electroosmotic laminar flow in a shear-driven outflow pathway was modeled by Navier–Stokes momentum equations for the velocity field, and Poisson’s equation for the electrical potential field. The hydrodynamic flow was modeled using the Navier–Stokes momentum equations with an electrical body force as follows:

(1) $$\rho \left(\frac{\partial u}{\partial t}+u\cdot \nabla u\right)=\mu \nabla^{2}u-\rho_{f}E$$

where *ρ* is the aqueous humor density, *u* is the velocity vector, *µ* is the aqueous humor viscosity, *ρ_f_* is the free charge density (charge per unit volume of the aqueous humor), and *E* is the induced electrical field vector which can be represented by the gradient of the electrical potential (*E =* −∇Φ). The electrical potential distribution was obtained using Poisson’s equation as $\nabla^{2}\Phi =-\frac{\rho_{f}}{\varepsilon }$ where *ε* is the dielectric permittivity (relative permittivity) of the aqueous humor in the outflow pathway.

The motion in the electroosmotic aqueous humor is induced by an applied electrical potential in the inlet of the outflow pathway where the TM surface facing the anterior chamber, which serves as the inlet to the outflow pathway. The aqueous humor flow causes stresses and strains in the outflow tissues through their active EFSI coupling algorithm. The mechanics of the outflow tissues can be represented as follows [68]:

(2) $$\nabla \cdot \left({\vec{V}}_{t}\right)=0$$

(3) $${\overset{=}{\sigma }}_{total}={\overset{=}{\sigma }}_{t}+{\overset{=}{\sigma }}_{i}$$

(4) $${\overset{=}{\sigma }}_{t}=\lambda e\overset{=}{I}+2\mu \overset{=}{E}$$

(5) $${\overset{=}{\sigma }}_{i}=-P_{i}\overset{=}{I}$$

(6) $$\nabla \cdot \left(\varepsilon_{t}{\overset{=}{\sigma }}_{total}\right)=0$$

where Equation (2) is the continuity equation assuming the existence of production and conversion for tissue. Assuming the presence of quasi-static conditions, and considerably small strain conditions, Equation (3) shows the stress tensor in tissue. Thus, *σ_total_*, *σ_t_*, *σ_i_* represent the total stress in the tissue, the internal stress in the tissue that is due to the elastic deformation of the outflow tissues, and the stress due to the presence and movement of the interstitial fluid in the outflow tissues that represent the hydraulic conductivity, respectively. Equation (4) that links the stress tensor and elastic deformation includes *λ* and *μ* as the Lame parameters, and *e* and *E* represent volumetric strain and strain tensor, respectively. Equation (5) represents the stress due to aqueous humor pressure and Equation (6) expresses the linear momentum equation in the quasi-static state. Considering the theory of infinitesimal strain, the following equations can be used:

(7) $$e=tr\left(\overset{=}{E}\right)=\nabla \cdot {\vec{u}}_{t}$$

(8) $$\overset{=}{E}=\frac{1}{2}\left(\nabla {\vec{u}}_{t}+\nabla \left({{\vec{u}}_{t}}^{T}\right)\right)$$

(9) $$\nabla \cdot \left({\vec{V}}_{t}\right)=\frac{\partial e}{\partial t}$$

where ${\vec{u}}_{t}$ represents the tissue displacement vector in units *m*.

The EFSI nonlinear partial differential equations were solved using COMSOL Multiphysics (COMSOL, Inc., Proprietary EULA, MA, USA). The methodology involved a segregated solution method in which the time-dependent solutions for the Poisson were obtained for the no-flow case in order to obtain the quiescent electric potential [69]. Aqueous humor was modeled to be homogeneous, laminar, Newtonian, and viscous [70], with the density and dynamic viscosity of 1000 kg/m^3^ (m refers to meter) and 0.7185 mPa·s (m refers to millimeter) [71], respectively. The zeta potential (the electrical potential at the slipping plane between the aqueous humor and the outflow tissues), electrical conductivity, and relative permittivity of the aqueous humor were specified as −19.5 mv (m refers to millimeter) [72], 179 × 10^−4^ (ohm^−1^ cm^−1^) [73], and 99 [74], respectively.

The simulation was conducted by a linear IOP elevation to from 0 to 15 mmHg (1 s) with the time step of 0.01 s (100 time steps) according to the physiological load rate [75,76]. The aqueous humor flow-out in the SC inner wall was modeled as open boundary with the boundary condition of normal stress (f_0_ = 0). Three different models were simulated, including the FSI with IOP of 15 mmHg, EFSI with IOP of 0 mmHg, and EFSI with IOP of 15 mmHg. While the EFSI case with an IOP of 0 mmHg is not physiologic, the aim was to calculate the role of the endothelial glycocalyx layer (electric) only on the biomechanics and hydrodynamics of the human aqueous outflow pathway. The EFSI simulations on average took ~32 h to run on our workstation. 

## 3. Results

**Figure 2:**
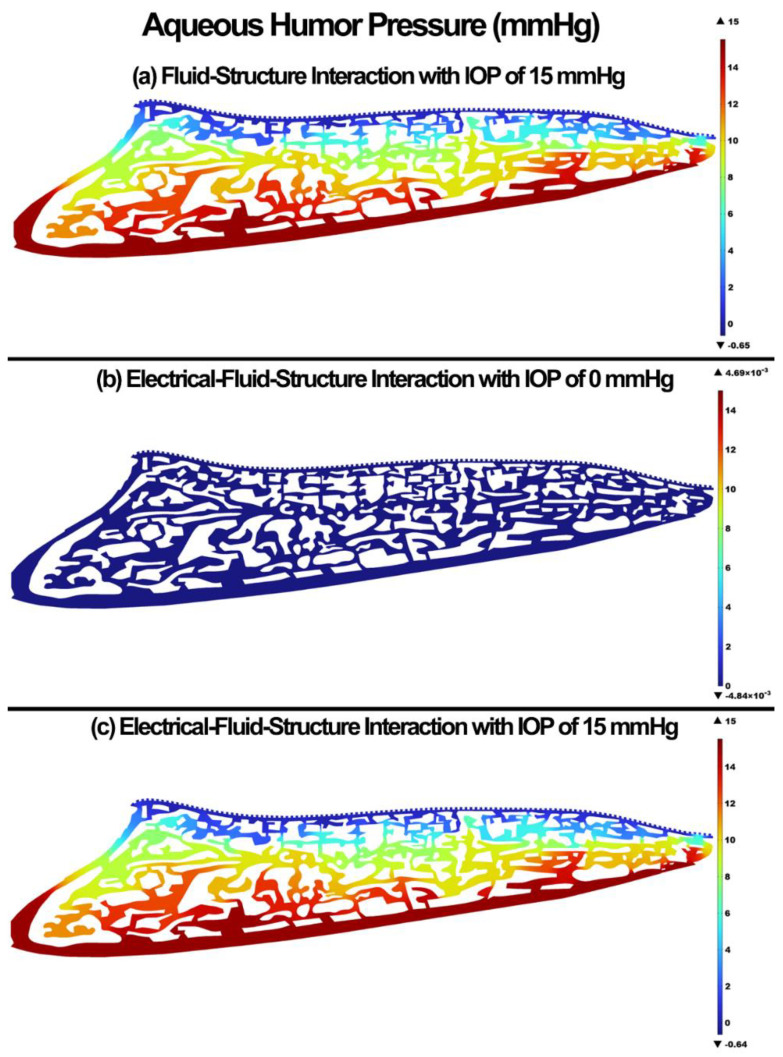
The aqueous humor pressure across the outflow pathway. (**a**) FSI model with IOP of 15 mmHg, (**b**) EFSI with IOP of 0 mmHg, and (**c**) EFSI with IOP of 15 mmHg.

**Figure 3:**
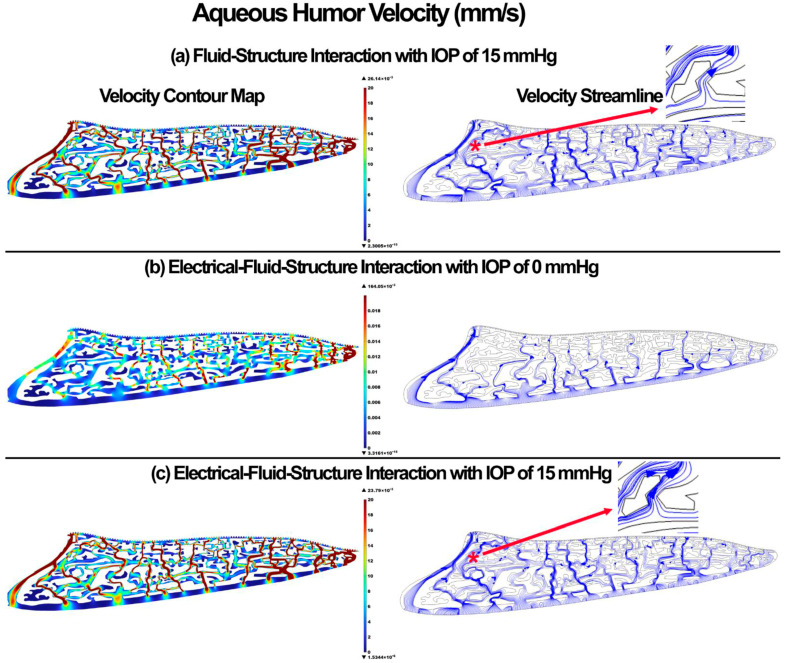
The aqueous humor velocity across the outflow pathway. (**a**) FSI model with IOP of 15 mmHg, (**b**) EFSI with IOP of 0 mmHg, and (**c**) EFSI with IOP of 15 mmHg.

The pressure and velocity in the aqueous humor for the FSI with IOP of 15 mmHg, EFSI with IOP of 0 mmHg, and EFSI with IOP of 15 mmHg are shown in Figure 2 and Figure 3, respectively. The resultant aqueous humor pressure and velocity in the outflow pathway were negligible at 0.00469 mmHg and 0.00164 mm/s, respectively. The pressure of velocity contour maps for both the FSI and EFSI models were relatively similar. However, the velocity streamlines across the outflow pathway showed relatively more tangential traction between the charged outflow tissues and aqueous humor when the role of the glycocalyx layers taken into account. Specific regions shown by a “*” show the role of the glycocalyx layer in providing a dynamic electrical relationship between the outflow tissue surfaces wall and the aqueous humor, and altering the aqueous humor hydrodynamics (inset).

**Figure 4:**
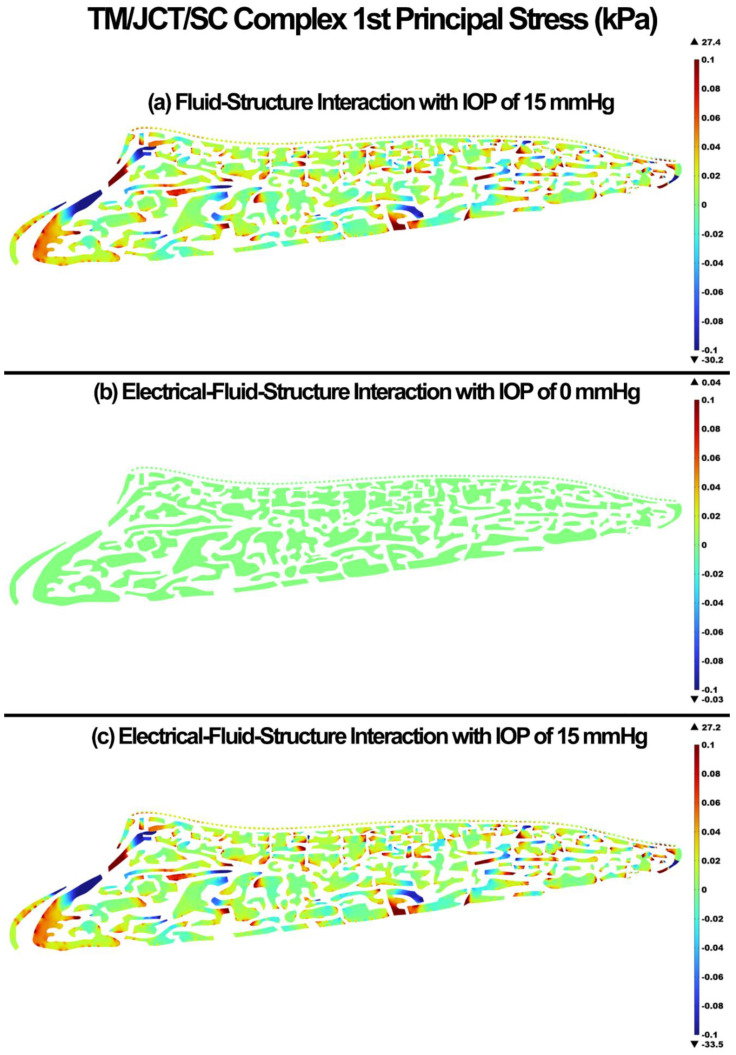
The 1st principal stress in the TM/JCT/SC complex. (**a**) FSI model with IOP of 15 mmHg, (**b**) EFSI with IOP of 0 mmHg, and (**c**) EFSI with IOP of 15 mmHg.

**Figure 5:**
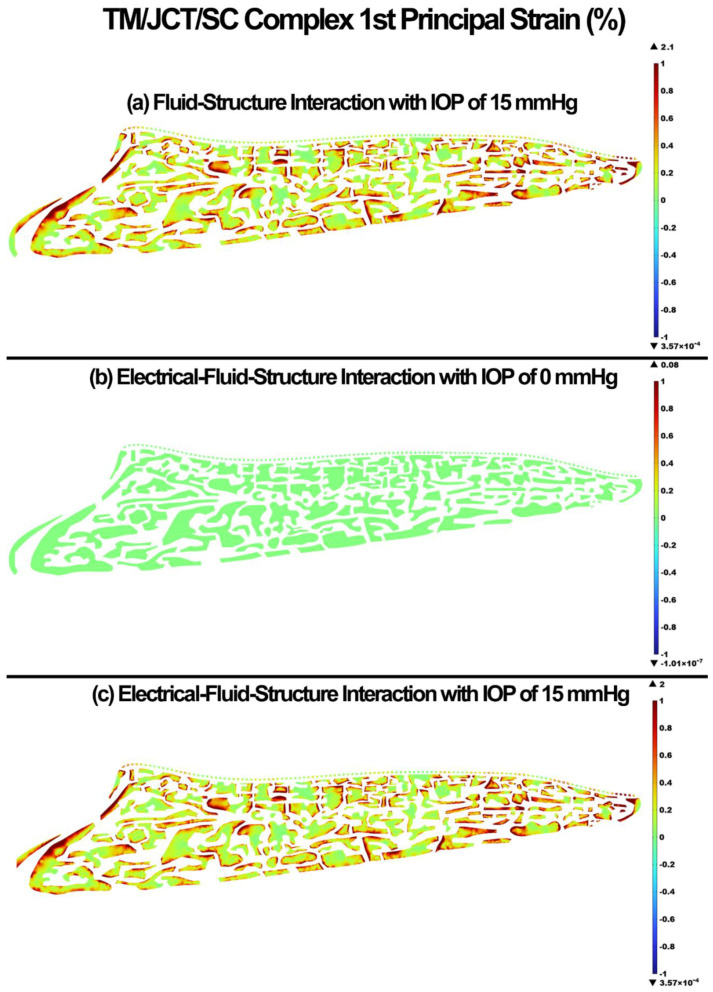
The 1st principal strain in the TM/JCT/SC complex. (**a**) FSI model with IOP of 15 mmHg, (**b**) EFSI with IOP of 0 mmHg, and (**c**) EFSI with IOP of 15 mmHg.

The 1st principal stress and strain in the TM/JCT/SC complex for the FSI with IOP of 15 mmHg, EFSI with IOP of 0 mmHg, and EFSI with IOP of 15 mmHg are shown in Figure 4 and Figure 5, respectively. The glycocalyx layer plays a minor role in the resultant stresses and strains in the outflow tissues. The EFSI models showed 0.2 kPa and 0.1% less tensile stress and strain, respectively, in the outflow tissues compared to the FSI model. 

**Figure 6:**
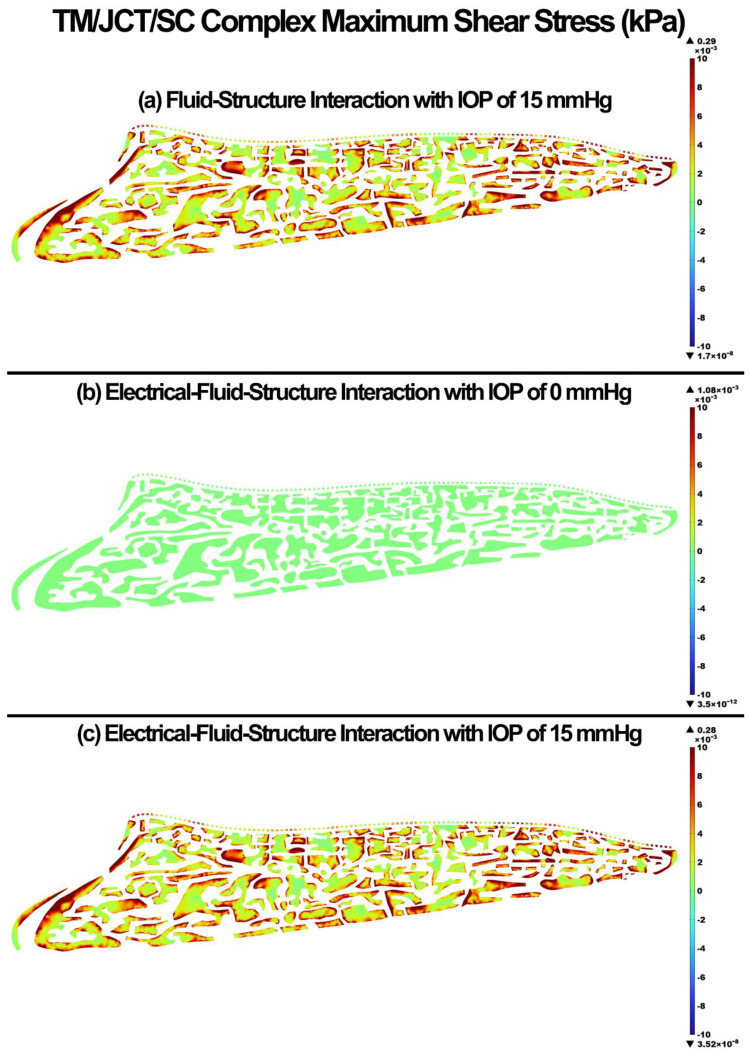
The maximum shear stress in the TM/JCT/SC complex. (**a**) FSI model with IOP of 15 mmHg, (**b**) EFSI with IOP of 0 mmHg, and (**c**) EFSI with IOP of 15 mmHg.

**Figure 7:**
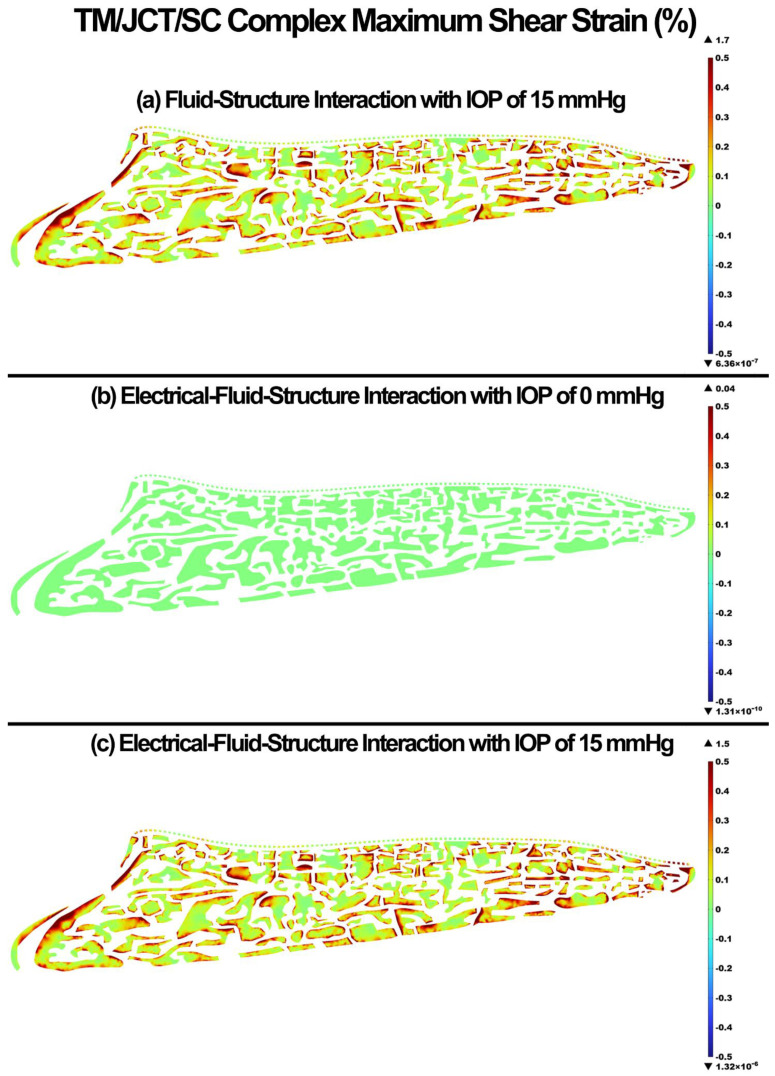
The maximum shear strain in the TM/JCT/SC complex. (**a**) FSI model with IOP of 15 mmHg, (**b**) EFSI with IOP of 0 mmHg, and (**c**) EFSI with IOP of 15 mmHg.

The maximum shear stress and strain in the TM/JCT/SC complex for the FSI with IOP of 15 mmHg, EFSI with IOP of 0 mmHg, and EFSI with IOP of 15 mmHg are shown in Figure 6 and Figure 7, respectively. The glycocalyx layer decreased the maximum shear stress and strain in the outflow tissues by 1 × 10^−4^ kPa and 0.2%, respectively.

**Figure 8:**
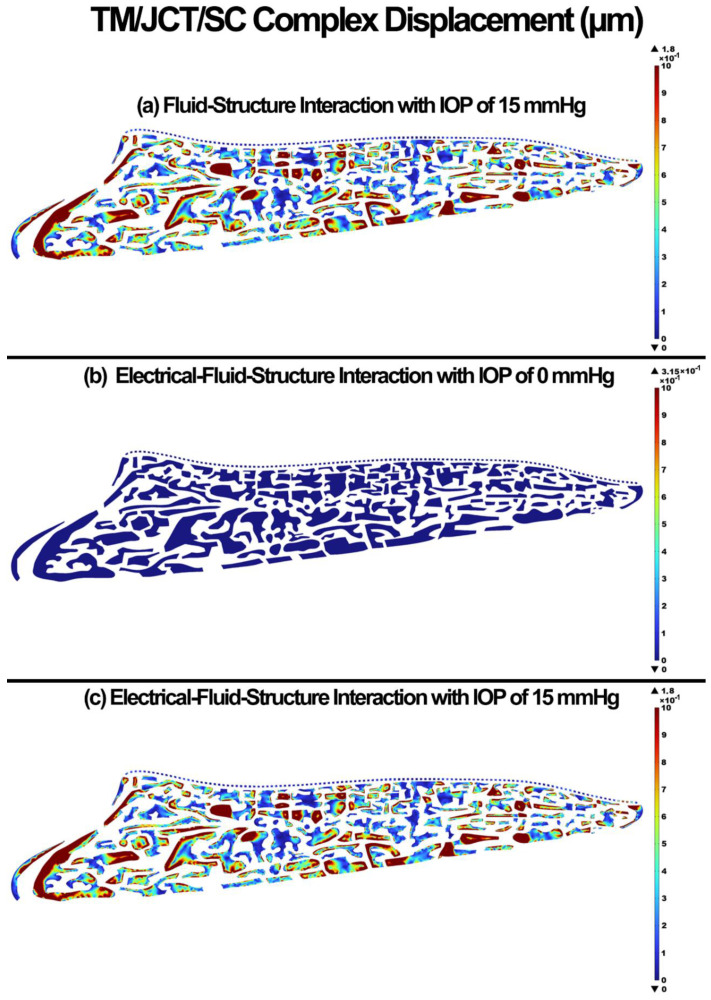
The displacement in the TM/JCT/SC complex. (**a**) FSI model with IOP of 15 mmHg, (**b**) EFSI with IOP of 0 mmHg, and (**c**) EFSI with IOP of 15 mmHg.

The displacement in the TM/JCT/SC complex for the FSI with IOP of 15 mmHg, EFSI with IOP of 0 mmHg, and EFSI with IOP of 15 mmHg are shown in Figure 8. The glycocalyx layer plays no role (~0 µm) in the resultant deformation of the outflow tissues.

## 4. Discussion

The cells in the outflow pathway are constantly exposed to fluctuating levels of fluid shear stress or shear induced mechanical forces [77] that results in actin cytoskeletal remodeling and changes the cell shape and mobility [24,27,28,29,30,31,77,78] as well as glycocalyx synthesis [79,80]. The glycocalyx layer has a significant contribution in transmitting the fluid shear stress to the cytoskeleton of endothelial cells [23,24,29,81,82,83] through the electroosmotic effect between the active interaction of the conductive aqueous humor and outflow tissues [44,45]. Since the glycocalyx functions as a mechanotransducer by influencing the resultant shear stress in the outflow tissues, as well as an activator of endothelial nitric oxide synthase (eNOS) with subsequent NO release, calculating the biomechanical role of the glycocalyx in an active EFSI interaction between the outflow tissues and aqueous humor is of great interest. While we are not aware of any experimental studies that have calculated the role of the glycocalyx layer in the resultant outflow resistance and aqueous humor hydrodynamics across the outflow pathway, numerical methods, such as the EFSI, may help to expand our knowledge of glycocalyx biomechanics. In this study, a 3D FE model of the human TM/JCT/SC complex was constructed [49,50] (Figure 1) and subjected to an aqueous humor inflow of 0 to 15 mmHg. The outflow tissues were covered with a thin electric double layer (~109 nm [19]) to represent the endothelial glycocalyx layer [51].

It has been experimentally shown that the glycocalyx layer may contribute to additional resistance against microvascular flow [84,85,86]. The in vivo flow resistance in small vessels was found about twice as large as the in vitro viscosity data, so the conclusion was that the endothelial glycocalyx layer is responsible for this discrepancy [84,85]. The endothelial glycocalyx layer in our study also caused a higher resistance for the flow, decreased the maximum aqueous humor velocity by 2.35 mm/s, and altered the streamlines or hydrodynamics of the flow across the outflow pathway (Figure 3); however, the profile of the pressure was unchanged (Figure 4). The aqueous humor through an active biomechanical interaction with the charged outflow tissues caused a shear-driven electroosmotic effect and caused a magnetic relationship between the flow and solid. This is why the streamlines of the velocity in the EFSI models tend to be closer to the wall compared to the FSI model (Figure 3 in inset regions).

The glycocalyx layer decreased the tensile stress and strain by 0.2 kPa and 0.1% in the outflow tissues, respectively (Figure 4 and Figure 5). The glycocalyx layer itself caused the stress and strain of 0.04 kPa and 0.08%, respectively, by pushing the aqueous humor into the outflow pathway. The same pattern was observed in the shear stress and strain (Figure 6 and Figure 7). The presence of the glycocalyx layer caused smaller shear stress and strain by 0.01 kPa and 0.2% in the outflow tissues, respectively, compared to the FSI model (no electrical interaction). The glycocalyx itself increased the shear stress and strain of 1.08 Pa and 0.04%, respectively, in the outflow tissues. This is in good agreement with Kapellos and colleagues [87], who showed that the presence of the glycocalyx causes negligible shear stress and physical forces acting on the outer surface of the tissues. The reduction in the resultant shear stresses and strains across the SC inner wall may affect NO release and IOP regulation as a result (Figure 6 and Figure 7). While the occluding junctions in the SC inner wall are very tight and can only account for a tiny fraction of conventional outflow [88], they maintain the apical-basal polarity of cells resulting in a steady electrical potential [89]. The glycocalyx layer also maintains the hydraulic resistance properties through a small pore system that does not permit the bulk flow of fluid across its boundaries [90]. Although the glycocalyx layer has a trivial biomechanical role in the outflow pathway, it still may affect NO synthesis, and in turn, the shear stress pattern in the outflow tissues (Figure 6). Nitric oxide is known as the vasodilator and relaxer in the TM that increases the outflow facility and decreases IOP [22]. Thus, even a small biomechanical contribution of the glycocalyx layer in the outflow pathway may play a role in the regulation of IOP [19]. The flow-induced shear stress in the blood vessels causes cell elongation and cell alignment in the direction of flow on the vascular endothelial cells [27,91,92]. The same pattern was observed in the conventional outflow pathway as even a relatively small shear stress in the SC aligns the endothelial cells with the flow [20]. This alignment is mediated by the glycocalyx, so the presence of the glycocalyx is essential for the endothelial cells to respond to fluid shear [28]. In the blood vessels, the glycocalyx is highly negatively charged layer that actively interacts with the plasma inducing several interfacial and mechanical as well as electrochemical phenomena [93]. In the outflow pathway, the negatively charged glycocalyx layer is known as a predominant reason why negatively charged red blood cells do not invade the vascular layers due to the electrostatic repulsion [94]. Similar to the blood vessels, in the human conventional outflow pathway, the glycocalyx may play a key role in maintaining the hydraulic resistance and maintaining a barrier to passage of proteins, like albumin [95].

Li showed the average TM displacements of ~1 µm at the IOP of 15 mmHg [96]. In our study, the nodal-averaged TM displacement in the FSI and EFSI models were ~0.59 and ~0.55 µm, respectively (Figure 8). Li [96] used nonhuman primate eyes (*Macaca nemestrina*) while we used human eyes, so the difference in TM displacements that we report could be due to species-related differences in biomechanical tissue properties.

### Limitations

First, the geometry of the JCT and SC inner wall and the µm-sized pores were manually selected in our models. While our FE model uses an idealized model for the outflow pathway, in future studies we will employ better optical coherence imaging techniques that allows us having eye-specific geometries for the JCT and SC inner wall.

Second, while it has been shown that the thickness of the glycocalyx across the human outflow pathway ranges from 52 to 166 nm [19], the thickest part is in the SC region, which may contribute to the higher outflow resistance noted there [21,97,98,99,100,101]. In the current study, the outflow tissues are covered with a uniform thickness of the glycocalyx (~109 nm). While this may affect the resultant outflow resistance in the outflow pathway, the aim of the present study was to develop a computational pipeline to model the role of the glycocalyx layer in the outflow resistance and IOP regulation. In our future studies, a localized thickness variation based on the available data in the literature will be defined in the outflow pathway. 

Third, the outflow tissues herein were treated as isotropic elastic materials based on properties that were measured using atomic force microscopy, while it has been shown that the tissues are anisotropic and viscoelastic [46,47,96,102]. While this may be considered as a limitation, the purpose of this study was to develop a computational approach to calculate the important role of the endothelial glycocalyx layer in the outflow resistance. Thus, the complex electrical equations for the glycocalyx layer were developed and coupled with the elastic solid and steady aqueous humor flow. In our future studies, we will extend the method, so we can address the active biomechanical interaction of the glycocalyx layer and aqueous humor dynamic with the viscoelastic outflow tissues. 

Fourth, we only modeled one normal eye, which does not represent the range of outflow tissues’ geometries present in the healthy population. Future studies will benefit from a larger cohort of healthy and glaucomatous eyes encompassing the accurate geometry of the tissues. 

Fifth, one may argue that changing the electrical properties of the outflow tissues and aqueous humor would affect the resultant stresses and strains in the outflow pathway. While we agree the electrical properties will affect the results, the available data in the literature for the aqueous humor and outflow tissues were used. Thus, with the current electrical properties, the resultant stresses and strains in the outflow tissues and hydrodynamics of the aqueous humor will not change. However, in future studies, each electrical parameter will be separately investigated to quantify their role in the resultant stresses and strains as well as the hydrodynamics in the outflow pathway.

Sixth, flow through the 360° circumference of TM structure is not uniform but divided into high- and low-flow regions, termed as segmental [9,12]. It has been shown that there is no significant difference between the thickness of the glycocalyx layer in the high- and low-flow regions [19], so herein, the segmental outflow was not modeled.

## 5. Conclusions

A thin electric double layer was applied to the surface of the 3D eye-specific FE model of a human conventional aqueous outflow pathway to represent the endothelial glycocalyx layer. The glycocalyx layer, outflow tissues, and the aqueous humor were coupled through the electrical–fluid–structure interaction and the model was subjected to IOP elevation from 0 to 15 mmHg. The electrical properties of the outflow tissues and the aqueous humor in a living eye can move the aqueous humor in the outflow pathway even without positive pressure in the anterior chamber. In the FSI model with no IOP (a non-physiologic model), the glycocalyx layer played a minor biomechanical role in the resultant stresses and strains, as well as the pressure and velocity of the aqueous humor. The findings of this study may have implications for understanding the biomechanical role of the endothelial glycocalyx layer. Calculating the resultant stresses and strains as well as the hydrodynamics of the aqueous humor in the human eye also provide quantitative information for clinicians, engineers, and pharmaceutical companies and provide a means of better understanding the complex electromechanical relationships in human eyes.

(a) The endothelial glycocalyx layer in the healthy human outflow pathway was modeled using EFSI.
(b) The positively charged aqueous humor can flow in the negatively outflow pathway with no IOP.
(c) The glycocalyx plays a minor role in the biomechanics and hydrodynamics of the outflow pathway.

## Data Availability

The raw/processed data required to reproduce these findings cannot be shared at this time as the data are part of an ongoing study.
